# Therapeutic roles of mesenchymal stem cell-derived extracellular vesicles in cancer

**DOI:** 10.1186/s13045-021-01141-y

**Published:** 2021-09-03

**Authors:** Zhijie Weng, Bowen Zhang, Chenzhou Wu, Fanyuan Yu, Bo Han, Bo Li, Longjiang Li

**Affiliations:** 1 State Key Laboratory of Oral Diseases, National Clinical Research Center for Oral Diseases, Department of Head and Neck Oncology, West China Hospital of Stomatology, Sichuan University, Chengdu, China; 2 State Key Laboratory of Oral Diseases, National Clinical Research Center for Oral Diseases, Department of Comfort Care Dental Center, West China Hospital of Stomatology, Sichuan University, Chengdu, China; 3 State Key Laboratory of Oral Diseases, National Clinical Research Center for Oral Diseases, West China Hospital of Stomatology, Sichuan University, Chengdu, China; 4 State Key Laboratory of Oral Diseases, National Clinical Research Center for Oral Diseases, Department of Orthodontics, West China Hospital of Stomatology, Sichuan University, Chengdu, China

**Keywords:** Mesenchymal stem cell, Extracellular vesicle, Exosome, Cancer therapy, Drug delivery

## Abstract

Extracellular vesicles (EVs) are cell-derived membrane structures enclosing proteins, lipids, RNAs, metabolites, growth factors, and cytokines. EVs have emerged as essential intercellular communication regulators in multiple physiological and pathological processes. Previous studies revealed that mesenchymal stem cells (MSCs) could either support or suppress tumor progression in different cancers by paracrine signaling via MSC-derived EVs. Evidence suggested that MSC-derived EVs could mimic their parental cells, possessing pro-tumor and anti-tumor effects, and inherent tumor tropism. Therefore, MSC-derived EVs can be a cell-free cancer treatment alternative. This review discusses different insights regarding MSC-derived EVs' roles in cancer treatment and summarizes bioengineered MSC-derived EVs’ applications as safe and versatile anti-tumor agent delivery platforms. Meanwhile, current hurdles of moving MSC-derived EVs from bench to bedside are also discussed.

## Background

Extracellular vesicles (EVs) are nano-sized bilayer-enclosed membrane structures containing proteins, lipids, RNAs, metabolites, growth factors, and cytokines, acting as versatile transporters between cells [1]. The EVs were first discovered by Peter Wolf in 1967 and were initially considered as “platelet dust” [2]. During the past 50 years, increasing information on EVs has become available. All cells can secrete EVs during normal and pathological processes [3]. EVs can participate in different diseases, especially cancers. EVs have been shown to transfer biomolecules between tumor cells, stromal cells, fibroblasts, endothelial cells, and immune cells, facilitating communication throughout the tumor microenvironment as paracrine mediators. Therefore, EVs are involved in cancer pathogenesis, progression, metastasis, and immunomodulation. The correlation between oncological states and EVs' existence in biological fluids favors their utility as an effective diagnostic tool in minimally invasive liquid biopsies by tumor biomarkers identification [4].

The more common application of EVs is based on their transport properties in delivering functional cargoes to targeted cells, rendering them attractive as drug delivery vehicles. Some native EVs harboring endogenous anti-tumor biomolecules can be exploited as therapeutic agents. Moreover, bioengineered EVs with additional desired cargoes and targeting specificity are holding brighter prospects in cancer therapy. Meanwhile, in contrast to other commonly applied drug delivery vehicles (e.g., liposomes), bioengineered EVs possess their merit due to their intrinsic targeting capabilities, low immunogenicity, high modification flexibility, as well as biological barrier permeability [5].

**Fig. 1:**
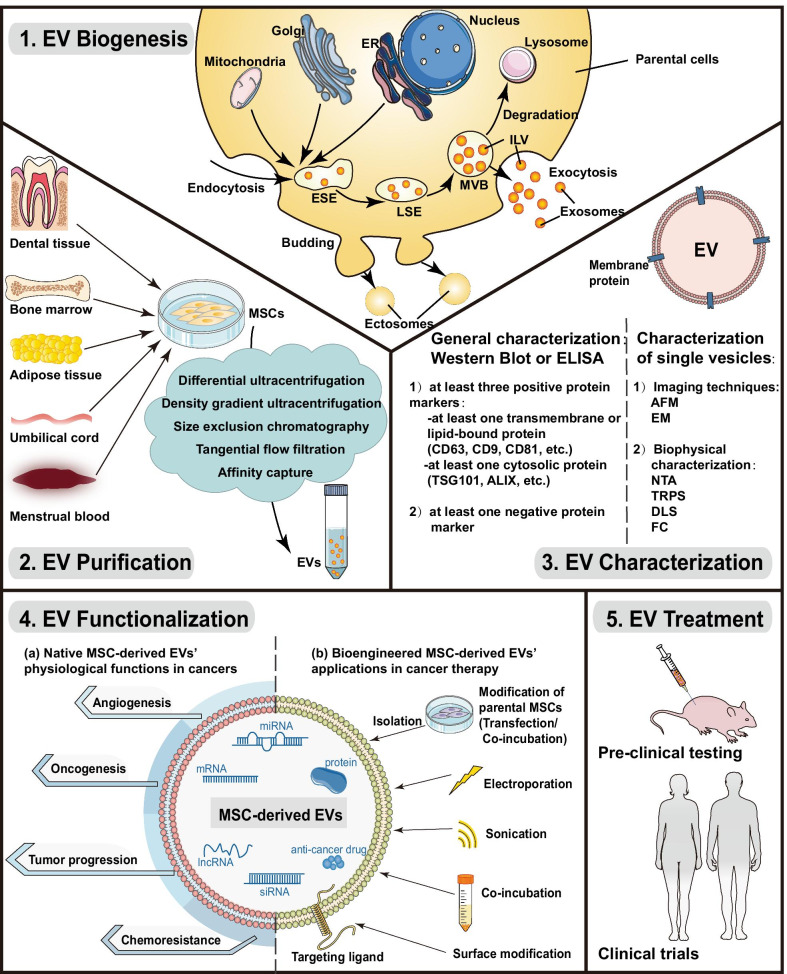
Key steps in the process of MSC-derived EVs’ therapeutic applications. *AFM* atomic force microscopy, *DLS* dynamic light scattering, *EM* electron microscopy, *ER* endoplasmic reticulum, *ESE* early-sorting endosome, *FC* flow cytometry, *ILV* intraluminal vesicle, *LSE* late-sorting endosome, *MVB* multivesicular body, *NTA* nanoparticle tracking analysis, *TRPS* tunable resistance pulse sensing

Since EVs are endogenous cellular products, they have an absolute requirement for parental cell sources to obtain prolific production. Nowadays, EVs for therapeutic applications are typically derived from mesenchymal stem cells (MSCs), a cell type well characterized for EV mass production [6]. MSCs, also known as multipotent mesenchymal stromal cells, are multipotent adult stem cells that can be isolated from bone marrow [7, 8], umbilical cord tissue [9, 10], placental tissue [11, 12], adipose tissue [13, 14], and dental tissue [15, 16]. Due to their potential to differentiate into mesoderm- and nonmesoderm-derived tissues, in vitro and in vivo [17], these cells can have a regenerative ability and are preferred for treating various tissue injuries. Besides, MSCs have been found to actively migrate toward inflammatory sites and to modulate immune responses [18]. Nevertheless, recently more attention has been focused on MSCs' therapeutic roles in cancer. MSCs can preferentially migrate toward tumors and be incorporated into tumor stroma [19–22]. Now it is well established that MSCs can regulate the tumor cell fate in a paracrine manner rather than a cellular one. MSC-derived EVs are major contributors among such paracrine effectors [23]. Moreover, MSC-derived EVs possess significant bioengineering potential as a guided anti-tumor drug delivery platform due to their strong migrating tropism toward tumor sites [24–26]. Figure 1 summarizes the key steps in the process of MSC-derived EVs’ therapeutic applications.

In this article, we first review EVs' biogenesis and their purification and characterization technologies. Then, we summarize current findings regarding MSC-derived EVs' physiological functions in cancers, from participation in tumor angiogenesis, proliferation inhibition and apoptosis promotion, to growth and metastasis facilitation, dormancy and chemoresistance induction. Next, we assemble the latest advances in drug loading and manufacturing of EV therapeutics, with particular emphasis on cargo and surface engineering techniques. MSC-derived EVs’ advantages as ideal drug delivery vehicles are also discussed by comparison with other nanocarriers and EVs derived from other sources. Then, based on the previous understanding, we detail the bioengineered MSC-derived EVs’ applications as a drug delivery system in cancer therapy. Finally, we discuss future challenges and directions regarding MSC-derived EV-based anti-cancer applications.

## Biological characteristics of extracellular vesicles

### Biogenesis

EVs are broadly categorized into two major classes: ectosomes and exosomes. Ectosomes (50–1000 nm in diameter) are vesicles released through plasma membrane outward budding and include microvesicles, microparticles, and large vesicles. Exosomes (40–160 nm in diameter) are endosomal vesicles formed through iterative plasma membrane invagination. After the early formation of cup-shaped structures, early-sorting endosomes (ESEs) and late-sorting endosomes (LSEs), multivesicular bodies (MVBs) are eventually generated, containing intraluminal vesicles (ILVs). Upon MVBs fusion with the plasma membrane, ILVs are released by exocytosis into the extracellular environment as exosomes. Some MVBs are degraded by lysosomes or autophagosomes fusion [4].

### Purification

Different technologies are currently used for EV purification, including differential ultracentrifugation, density gradient ultracentrifugation, size exclusion chromatography, tangential flow filtration, and affinity capture [27].

Until now, differential ultracentrifugation—an initial, well-established, and reliable method—is still the most widely adopted approach due to its simplistic protocol and relatively high yield [28]. By increasing centrifugation speed and/or time in a stepwise manner, it can separate particles with different sedimentation rates, then remove undesired components during each centrifugation. However, this approach cannot distinguish particles with overlapping ranges, such as exosomes and microvesicles. Density gradient ultracentrifugation, size exclusion chromatography, and filtration present similar problems, depending on particle density or size for separation. Different from these physical-based isolation methods, affinity capture can separate EVs with high-purity but with low-yield via EV surface markers interaction with the capture molecules attached to different carriers (e.g., magnetic beads) [29].

The International Society for Extracellular Vesicles (ISEV) has proposed detailed guidance for these isolation methods [30]. However, none achieved the absolute purification, that is, completely isolating EVs from other biological products. Each method has advantages and disadvantages, and their combinations might be recommended for maximum EV enrichment. Based on some comparative studies [31–35], we have summarized the characteristics of different EV isolation methods in Table 1.

**Table 1:** Overview of EV isolation methods

Isolation method	Principle	Advantages	Disadvantages	References
Differential ultracentrifugation	Based on differential centrifugation	Most commonly used and well established Simple Economical Relatively high yield	Low purity Low upscale potential	[31, 34]
Density gradient ultracentrifugation	Based on density gradient of solutions	Relatively high purity Maintain EV integrity	Time-consuming Lower yield Low upscale potential	[31, 34]
Size exclusion chromatography	Based on particle size	Economical Relatively high purity Maintain EV integrity High upscale potential	Time-consuming Lower yield Contamination	[32]
Tangential flow filtration	Based on particle size	High yield High purity High time-efficiency High upscale potential	Complicated equipment Difficult operation Limited understanding	[33, 35]
Affinity capture	Based on interaction of capture molecule with EV antigen	High purity Specific separation	Low yield Costly Separate targeted proteins only	[31, 34]

### Characterization

It is essential to thoroughly characterize EVs according to ISEV’s minimal criteria report to validate the isolation method. A comprehensive EV characterization embraces general and single vesicle characterization.

The general characterization usually focuses on some protein markers using Western Blot or ELISA. The ISEV suggests the characterization of at least three positive and one negative EV protein marker. Positive protein markers should include at least one transmembrane/lipid-bound protein (e.g., CD63, CD9, CD81) and one cytosolic protein (e.g., TSG101, ALIX).

Single vesicle characterization requires imaging techniques and biophysical characterization. Atomic force microscopy (AFM) and electron microscopy (EM), including transmission electron microscopy (TEM) and scanning electron microscopy (SEM), are the only imaging techniques able to capture high-resolution EV morphology images. Immunogold EM is commonly used to stain specific EV markers. Biophysical characterization involves nanoparticle tracking analysis (NTA), tunable resistance pulse sensing (TRPS), dynamic light scattering (DLS), and flow cytometry (FC), for example [30].

However, detailed characterization of EV subpopulations and molecular composition of each EV type remains unavailable [36].

## Physiological functions of MSC-derived EVs in cancers

### Participation in tumor angiogenesis

Discussions about MSC-derived EVs’ functions in cancer emerged since Zhu et al. [37] firstly reported that exosomes secreted by MSCs could promote tumor growth in vivo, similarly to MSCs. They found that exosomes derived from human bone marrow mesenchymal stem cells (hBMSCs) favored tumor growth in xenograft mice models of gastric and colon cancers. However, the exosomes did not present similar effects on tumor cells in vitro. On the other hand, angiogenesis-related molecular signaling pathway activation was found in vivo and in vitro with increased VEGF and CXCR4 mRNA levels, which coincided with the higher vascular density observed in tumor tissues in vivo. Finally, they demonstrated that hBMSC-derived exosomes could increase VEGF and CXCR4 expression in tumor cells by ERK1/2 and p38 MAPK pathways activation, leading to enhanced angiogenesis, thus promoting tumor growth in vivo.

However, opposite effects have been discovered in breast cancer cells. Lee et al. [38] reported that hBMSC-derived exosomes could inhibit angiogenesis and tumor progression in vitro and in vivo by transferring miR-16 into tumor cells, which could target VEGF and reduce its expression in breast cancer cells. They were the first to describe tumor microenvironment reprogramming conducted by miRNAs in MSC-derived exosomes. This view was supported by Pakravan et al. [39], who pointed out that miR-100 was enriched in hBMSC-derived exosomes and suppressed angiogenesis in vitro through VEGF downregulation in breast cancer cells. Further, they demonstrated that miR-100 exosomal transfer mediated VEGF expression via the mTOR/HIF-1α signaling axis.

Besides BMSCs, human menstrual stem cells (MenSCs) isolated from menstrual fluids also have great potential as angiogenic regulators. It is easy to understand based on common sense that physiological angiogenesis occurs during the female menstrual cycle. Currently, the exploration of MenSCs’ therapeutic mechanisms is only emerging, especially in the cancer context. Alcayaga-Miranda et al. found that MenSC-derived exosomes decreased angiogenesis in prostate adenocarcinoma in vivo and in vitro, inhibiting reactive oxygen species (ROS) pathway, therefore downregulating the secretion of pro-angiogenic factors (e.g. VEGF, FGF) and NF-κB transcription factor [40]. Besides, by altering prostate adenocarcinoma cell culturing conditions, they successfully proved that the observed anti-angiogenic effect was mediated by exosomes rather than direct intercellular contact with MenSCs or other secretomes. Also, tumor angiogenesis and growth inhibition was found in the hamster buccal pouch carcinoma model treated with MenSC-derived exosomes [41]. In this paper, tumor cells and endothelial cells internalized MenSC-derived exosomes and had lower VEGF expression under exosomal modulation, resulting in tumor angiogenesis and growth inhibition in vivo.

### Proliferation inhibition and apoptosis promotion

Despite MSC-derived EVs’ indirect pathway to modulate tumor angiogenesis that influences tumor growth in turn, many researchers tried to clarify whether MSC-derived EVs can directly affect tumor cell proliferation and apoptosis in cancer progression. In the beginning, researchers used different cancer cell lines and mice xenograft models to verify MSC-derived EVs’ modulatory roles in the cancer cell cycle, proliferation, and apoptosis. EVs from hBMSCs have been reported to activate cell cycle negative regulators, leading to apoptosis or necrosis and anti-proliferation of tumor cells in hepatocellular carcinoma, ovarian cancer, and Kaposi’s sarcoma [42]. Similarly, the anti-proliferative and pro-apoptotic effects of EVs derived from human umbilical cord mesenchymal stem cells (hUCMSCs) were detected in bladder carcinoma. These effects were related to restrained AKT protein kinase phosphorylation and increased Caspase 3 cleavage [43].

Next, continued concern has been raised about which factor delivered by MSC-derived EVs into target tumor cells were dominant in cancer progression. Reza et al. [44] observed that incorporating human adipose mesenchymal stem cell (hAMSC)-derived exosomes attenuated ovarian cancer cell proliferation and induced apoptosis. Next, they treated ovarian cancer cells with protease-digested exosomes or RNase-digested exosomes to explore whether exosomal protein or RNA was responsible for the observed effects. No significant differences between protease-digested and fresh exosomes were detected, while the RNase-digested exosomes had no anti-proliferation effect in ovarian cancer cells. After subsequent verifications, they concluded that oncogene-related miRNAs in hAMSC-derived exosomes were responsible for the anti-tumor activities observed. The miRNAs led to enhanced mitochondria-mediated apoptosis in ovarian cancer cells by pro-apoptotic molecules upregulation and anti-apoptotic proteins downregulation.

To date, researchers have gained a better understanding of miRNAs in different MSC-derived EVs in various cancer types. For instance, miRNA-145 upregulation in hAMSC-derived exosomes had a suppressive role in prostate cancer progression and induced apoptosis via the Caspase-3/7 pathway [45]. Another miRNA, let-7i, could be transferred from hBMSC-derived EVs into lung cancer cells to abolish tumor cell proliferation via the KDM3A/DCLK1/FXYD3 axis [46]. However, further extensive investigations are still required to determine the underlying mechanism of exosomal miRNAs or other unknown cargoes in cancer progression.

### Growth and metastasis facilitation

On the other hand, MSC-derived EVs can also exhibit pro-proliferative effects on cancer cells, different from their described roles so far. For example, hBMSC-derived EVs promoted proliferation, migration, and tumorigenesis in nasopharyngeal carcinoma [47] and osteosarcoma [48]. HUCMSC-derived EVs had a similar effect in renal cancer [49], lung cancer [50, 51], and breast cancer [52]. It is not surprising that miRNAs contained in EVs have also been verified as important contributors to such modulations. For example, transferred miR-410 from hUCMSC-derived EVs favored lung adenocarcinoma growth by targeted inhibition of PTEN, which was involved in tumor cell proliferation and apoptosis [50]. Another miRNA, miR-130b-3p, was also enriched in hUCMSC-derived EVs and transferred into lung cancer cells, playing an oncogenic role via the FOXO3/NFE2L2/TXNRD1 axis [51]. Likewise, overexpressed miR-21-5p, delivered by hypoxia pre-challenged hBMSC-derived EVs, exerted pro-proliferative and pro-metastatic effects by abrogating apoptosis and inducing macrophage M2 polarization in lung cancer, with low protein expression of several pro-apoptotic genes (e.g., PTEN, PDCD4, and RECK)[53]. Also, lower-expressed miR-15a in hBMSC-derived exosomes from multiple myeloma patients was identified as a key mediator in pro-tumor activities [54].

Additionally, lncRNAs, mRNAs, and proteins encapsulated in EVs received increasing attention. Du et al. [49] reported that hUCMSC-derived EVs promoted tumor growth and metastasis in renal cancer via AKT and ERK1/2 signaling pathways activation. The effect was derived from hepatocyte growth factor (HGF) synthesis induction in the presence of human HGF mRNA transferred by the EVs. Zhao et al. [48] demonstrated that the lncRNA PVT1 packed in hBMSC-derived exosomes up-regulated the oncogenic protein ERG by restraining ERG degradation and ubiquitination, as well as sponging miR-183-5p. Finally, it brought about enhanced growth and metastasis in osteosarcoma. Regarding exosomal proteins, Mao et al.[55] reported that E3 ubiquitin-protein ligase UBR2 was enriched in p53 deficient mouse BMSC-derived exosomes. UBR2 expression was also increased in gastric cancer cells treated with the exosomes, enhancing tumor growth and metastasis via the Wnt/β-catenin pathway. Overall, these results suggested that exosomal miRNAs, lncRNAs, mRNAs, and proteins can be transported into target cells and play specific roles.

Particularly, vital links between epithelial-mesenchymal transition (EMT) and tumor progression received growing recognition. EMT is a cellular process in which cells switch from an epithelial phenotype to a mesenchymal one, reducing cell-to-cell adhesion and elevating migratory capacity [56]. Several studies have shown that the MSC-derived EVs’ pro-metastatic effects in tumor cells are related to EMT induction. Shi et al. [47] discovered that hBMSC-derived exosomes enhanced FGF19-FGFR4 dependent ERK signaling cascade activation and induced EMT of nasopharyngeal carcinoma cells. They were incubated with the exosomes, leading to enhanced tumor growth and metastasis. Similarly, Zhou et al. [52] reported that hUCMSC-derived EVs facilitated tumor progression and metastasis in breast cancer by EMT induction via ERK pathway upregulation.

### Dormancy and chemoresistance induction

Tumor dormancy has been a research hotspot in metastatic cancer progression. It refers to tumor cells' ability to remain in small amounts and undetectable at the metastatic site after primary tumor resection. The dormancy is associated with chemoresistance, prolonged asymptomatic residual disease, and cancer recurrence [57]. Breast cancer is one of the best-known tumor dormancy cases. Disseminated breast cancer cells can migrate to the bone marrow, then induce prolonged dormancy within the mesenchymal stem cell niche, down-regulating cell proliferation and invasion, as well as up-regulating cell adhesion [58]. Questions have been raised about the dormancy initiation in the bone marrow microenvironment. Evidence suggested that the resident MSCs play a key role [59]. Therefore, researchers have focused on involved cellular mechanism between MSCs and tumor dormancy. Ono et al. [60] demonstrated that exosomes secreted from hBMSCs transferred miR-23b into metastatic breast cancer cells, inducing tumor dormancy by inhibiting its target oncogene MARCKS. This finding was consistent with Casson et al. [61]. Casson et al. reported that metastatic breast cancer cells treated with hBMSC-derived EVs were induced to undergo a mesenchymal–epithelial transition (MET) and maintained a dormant state, shown as migration inhibition and cell adhesion promotion. The two studies showed that the dormancy kept tumor cells in a cycling quiescent state, thus helping them hide from chemotherapy and gain chemoresistance.

Similarly, hUCMSC-derived exosomes enforced dormancy and protected tumor cells against conventional treatments by transferring exosomal miRNAs in metastatic breast cancer [62]. Apart from breast cancer, gastric cancer's chemoresistance was also enhanced by hUCMSC-derived exosomes [63]. In this case, exosomal proteins, rather than exosomal miRNAs, conferred the drug resistance by CaM-Ks/Raf/MEK/ERK pathway activation.

**Fig. 2:**
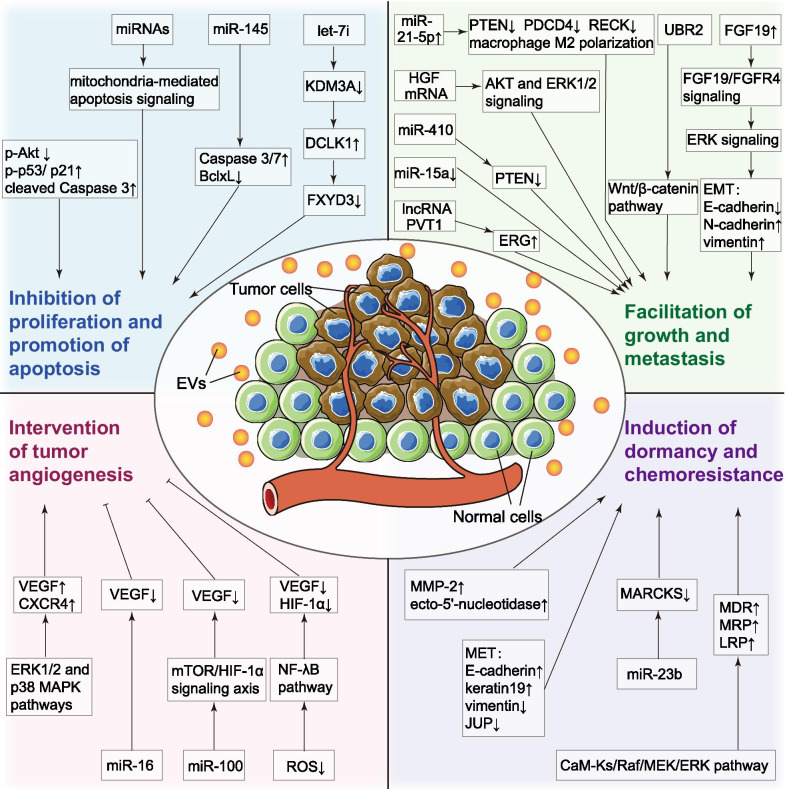
Physiological functions of MSC-derived EVs in cancers

In conclusion, EVs derived from different MSCs have diverse effects on specific tumors. The studies mentioned in this section are summarized in Table 2 and Fig. 2. These conflicting experimental results could be associated with the heterogeneity of MSCs, the complexity of tumor microenvironment, the diversity of malignancies’ origin, and the difference of experimental conditions. Multiple mechanisms and cargoes of the EVs may be involved in tumor progression modulation. There is still ample room for further progress to articulate these signaling interactions.

**Table 2:** Effects of native MSC-derived EVs on different types of cancer

EV source	Cancer	Method	Key cargo	Effect	Proposed mechanism	Reference
hBMSCs	Gastric cancer; colon cancer	In vitro and in vivo	N/A	Angiogenesis↑ Cell proliferation↑	Activation of ERK1/2 and p38 MAPK pathways	[37]
hBMSCs	Mouse breast cancer	In vitro and in vivo	miR-16	Angiogenesis↓ Tumor progression↓	VEGF↓	[38]
hBMSCs	Breast carcinoma	In vitro	miR-100	Angiogenesis↓ Endothelial cell proliferation↓ Migration↓	mTOR/HIF-1α/VEGF signaling axis	[39]
hMenSCs	Prostate adenocarcinoma	In vitro and in vivo	N/A	Angiogenesis↓ Tumor progression↓	ROS↓ VEGF↓	[40]
hMenSCs	Hamster buccal pouch carcinoma	In vitro and in vivo	N/A	Endothelial cell apoptosis↑ Tumor progression↓	VEGF↓	[41]
hBMSCs	Hepatocellular carcinoma; ovarian cancer; Kaposi’s sarcoma	In vitro and in vivo	N/A	Tumor progression↓	Activation of negative regulators of cell cycle	[42]
hUCMSCs	Bladder carcinoma	In vitro and in vivo	N/A	Proliferation↓ Apoptosis↑	Phosphorylation of Akt protein kinase↓ p53/p21 and Caspase 3↑	[43]
hAMSCs	Ovarian cancer	In vitro	miRNAs	Proliferation↓	Activation of mitochondria-mediated apoptosis signaling	[44]
hAMSCs	Metastatic prostate cancer	In vitro and in vivo	miR-145	Proliferation↓ Apoptosis↑	BclxL↓	[45]
hBMSCs	Lung cancer	In vitro and in vivo	let-7i	Proliferation↓ Metastasis↓	KDM3A↓ DCLK1↑ FXYD3↓	[46]
hBMSCs	Nasopharyngeal carcinoma	In vitro and in vivo	N/A	Proliferation↑ Migration↑ Tumorigenesis↑	FGF19-FGFR4 dependent ERK signaling cascade; EMT	[47]
hBMSCs	Osteosarcoma	In vitro and in vivo	lncRNA PVT1	Tumor growth↑ Metastasis↑	Stabilize ERG and sponge miR-183-5p	[48]
hUCMSCs	Renal cancer	In vitro and in vivo	HGF mRNA	Tumor growth↑ Aggressiveness↑	Activation of AKT and ERK1/2 signaling	[49]
hUCMSCs	Lung adenocarcinoma cancer	In vitro and in vivo	miR-410	Proliferation↑ Apoptosis↓	PTEN↓	[50]
hUCMSCs	Lung cancer	In vitro and in vivo	miR-130b-3p	Proliferation↑ Migration and invasion↑ Apoptosis↓	FOXO3↓ Activation of NFE2L2/TXNRD1 pathway	[51]
hUCMSCs	Breast cancer	In vitro	N/A	Proliferation↑ Migration and invasion↑	Induction of EMT via the ERK pathway	[52]
hBMSCs	Non-small cell lung cancer	In vitro and in vivo	Increased miR-21-5p	Tumor growth↑ Proliferation↑ Invasion↑	Macrophage M2 Polarization	[53]
hBMSCs of patients with multiple myeloma	Multiple myeloma	In vitro and in vivo	Lower miR-15a	Tumor growth↑ Dissemination↑	Oncogenic proteins, cytokines, and adhesion molecules↑	[54]
p53 deficient mBMSCs	Mouse gastric cancer	In vitro and in vivo	UBR2	Tumor growth↑ Metastasis↑ Stemness↑	Abnormal activation of Wnt/β‐catenin signaling pathway	[55]
hBMSCs	Bone marrow–metastatic breast cancer	In vitro and in vivo	miR-23b	Proliferation and invasion↓ Sensitivity to docetaxel↓ Dormancy↑	MARCKS↓	[60]
hBMSCs	Breast carcinoma	In vitro	N/A	Proliferation and migration↓ Dormancy↑ Anti-cancer drug resistance↑	MET	[61]
hUCMSCs	Breast cancer; ovarian cancer	In vitro	N/A	Tumor heterogeneity↑ Dormancy↑ Tumor cell resistance↑	Induction of MMP-2 and ecto-5′-nucleotidase activity	[62]
hUCMSCs	Gastric cancer	In vitro and in vivo	Proteins	Drug resistance↑	CaM-Ks/Raf/MEK/ERK signaling cascade↑	[63]

*EV* extracellular vesicle, *hAMSCs* human adipose mesenchymal stem cells, *hBMSCs* human bone marrow mesenchymal stem cells, *hMenSCs* human menstrual stem cells, *hUCMSCs* human umbilical cord mesenchymal stem cells, *mBMSCs* mouse bone marrow mesenchymal stem cells, *MSC* mesenchymal stem cell

## Current technologies for drug loading and manufacturing of EV therapeutics

**Fig. 3:**
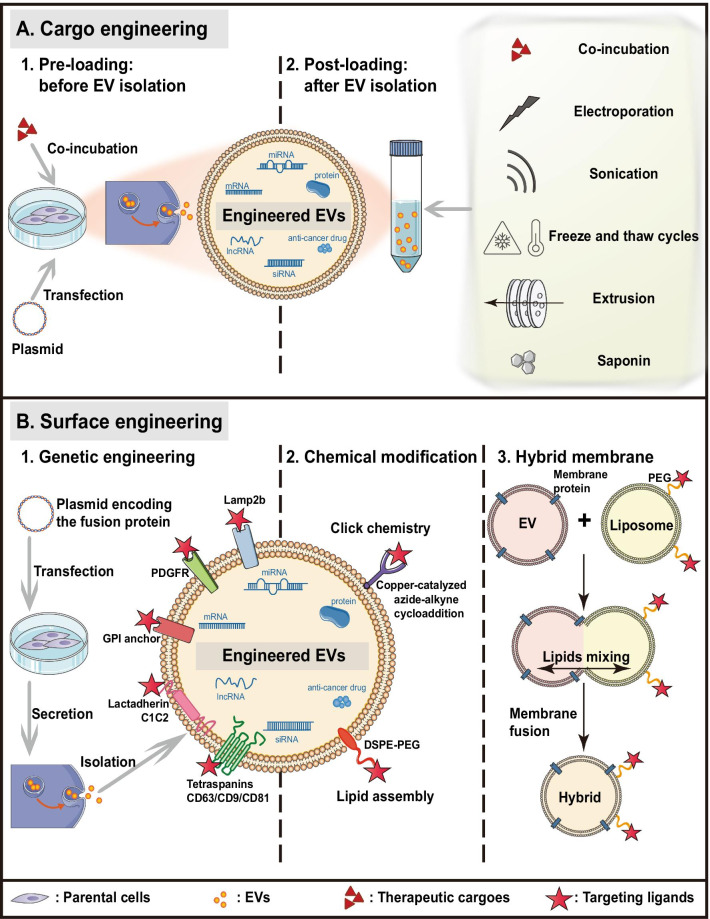
Current technologies for EV bioengineering. EV bioengineering technologies are generally divided into two categories: cargo engineering (**A**) and surface engineering (**B**). *DSPE* 1,2-distearoyl-sn-glycero-3-phosphoethanolamine, *GPI* glycosylphosphatidylinositol, *Lamp2b* lysosomal-associated membrane protein 2, *PDGFR* platelet-derived growth factor receptor, *PEG* polyethylene glycol

Compared with native EVs, bioengineered EVs exhibit a higher therapeutic potential as delivery vehicles because they can transfer desired cargoes and confer enhanced targeting specificity. So far, two major strategies are applied to maximize therapeutic efficacy of EVs: cargo engineering and surface engineering (summarized in Fig. 3).

### Cargo engineering

EVs can encapsulate different therapeutic agents, including drugs, proteins, and nucleic acids. Cargo loading approaches are generally divided into two categories: pre-loading (before EV isolation) and post-loading (after EV isolation).

#### Pre-loading

By modifications of parental cells, therapeutic cargoes can be endogenously packaged into EVs during the biogenesis process before EV isolation. This can be performed by genetic manipulation of parental cells [64]. By cell transfection, parental cells can overexpress therapeutic miRNAs, siRNAs, mRNAs, proteins, and peptides, which will subsequently be encapsulated into EVs. Another approach is directly incubating drugs with parental cells, enabling the production of drug-containing EVs.

Pre-loading strategies provide relatively simple and stable production of EVs enclosed with desired active components, besides maintaining EV membrane integrity. However, they are time-consuming and have low efficiency, typically leading to limited loading potential.

#### Post-loading

The post-loading occurs after EV isolation. The exogenous cargoes are encapsulated into EVs by passive loading or active loading.

Hydrophobic drugs can be combined with the EV lipid bilayer membrane after direct co-incubation, attaching to the EV surface. This passive loading strategy depends on the molecules’ concentration gradient and the cargoes' hydrophobic nature, usually leading to a low loading capacity [65].

Regarding hydrophilic drugs, different active loading strategies have been proposed to temporarily permeabilize the hydrophobic lipid membrane, physically or chemically, allowing the diffusion of drugs into EVs. Physical approaches—such as electroporation, sonication, freeze and thaw cycles, extrusion—generally involve transient disruption of EV membrane by external forces [5]. Currently, electroporation is the most used one, especially for RNA encapsulation. Differently, chemical approaches utilize transfection reagents, or permeabilizers, such as saponin, to facilitate cargoes' entrance into the EV without destroying its lipid bilayer structure [66].

Each strategy has its advantages and limitations (summarized in Table 3). Overall, caution is required to avoid EV aggregation, EV membrane damage or immunogenicity induction during post-loading procedures [67–73].

**Table 3:** Advantages and disadvantages of post-loading methods

Post-loading method	Category	Advantages	Disadvantages	References
Direct co-incubation	Passive loading	Simple Maintain EV membrane integrity	Low loading efficiency Time-consuming Limited cargo range	[69, 71]
Electroporation	Physically-induced active loading	High loading efficiency	SiRNA aggregation Potential influence of stability	[70]
Sonication	Physically-induced active loading	High loading efficiency	Disrupt EV membrane integrity Damage cargoes	[68, 72]
Freeze and thaw cycles	Physically-induced active loading	Medium loading efficiency	EV aggregation	[73]
Extrusion	Physically-induced active loading	High loading efficiency	Disrupt EV membrane integrity	[68]
Saponin/chemical transfection	Chemically-induced active loading	High loading efficiency	Immunogenetic toxicity Potential influence of stability	[66]

#### Novel technologies for cargo loading

Recently, an optically reversible protein–protein interaction (EXPLORs) technology has been reported to encapsulate anti-inflammatory proteins into EVs [74]. In this case, cargo proteins are fused with the photoreceptor cryptochrome 2 (CRY2), and the basic-helix-loop helix 1 (CIB1) protein is fused with the EV surface protein CD9. CRY2 can bind with CIB1 under blue light irradiation (at 460 nm), allowing the cargo packaging into EVs.

Additionally, some RNA binding proteins on the EV surface have been explored, such as Y-box protein 1 [75], ELVA protein HuR [76], and hnRNPA2B1 [77]. They can enable the specific loading of therapeutic RNAs into EVs.

### Surface engineering

EVs derived from different cell sources have various surface molecules, displaying selectivity for specific recipient cells. Altering the surface of EVs, especially protein composition, can alter the biodistribution and tropism of EVs. The main goal of surface engineering is to endow EVs with additional targeting specificity, thereby increasing the local concentration of EVs at desired sites, reducing unwanted systemic toxicity. Surface engineering technologies can be classified into three categories: genetic engineering, chemical modification, and hybrid membrane engineering.

#### Genetic engineering

EVs have native transmembrane proteins that can be modified with exogenous targeting ligands. Genetic engineering is a valid method for displaying a targeting ligand on the EV membrane surface by parental cells transfection with plasmids encoding the fusion protein of the targeting ligand and the selected EV transmembrane protein. Alternatively, the targeted epitope can also be inserted into the desired protein domain, instead of fusing with the whole protein [65].

Lysosomal-associated membrane protein 2 (Lamp2b), enriched in dendritic cell-derived exosomes, was the first reported and is the most widely used exosomal membrane protein in surface engineering approaches [78]. The N-terminus of Lamp2b is displayed on exosome surface and can be appended with different targeting ligands. For instance, the neuron-specific peptide rabies viral glycoprotein (RVG) [78], αγ integrin-specific peptide iRGD [79], and HER2-binding affibody zHER [80] have been anchored on EVs through fusion with Lamp2b to impart EVs with selective migration toward the central nervous system, integrin-positive breast cancer cells, and HER2-expressing tumor cells separately.

Despite these successes, a major limitation is that Lamp2b-inserted peptides are vulnerable to endosomal protease degradation. To improve the long-term stability of Lamp2b hybrids, a glycosylation motif (GNSTM) can be added to the peptide-Lamp2b fusions [81]. Besides, other membrane protein candidates have been investigated, such as the platelet-derived growth factor receptor (PDGFR) transmembrane domain [82], glycosylphosphatidylinositol (GPI) anchor peptides [83], the lactadherin C1C2 domain [84], and the tetraspanin superfamily CD63/CD9/CD81 with their two extracellular loops [76, 85, 86]. They all exhibit excellent performance for functional ligand bearing with high binding affinity and selectivity to target tissues. However, such strategies are often time-consuming and challenging due to the complex manipulation of parental cells. They can also cause immune activation and functional losses of host proteins.

#### Chemical modification

Targeting ligands can also be attached to the EV surface by chemical modification after EV isolation, relying on bioconjugation reactions or lipid assembly.

Click chemistry is a representative technology that realizes the bioconjugation of targeting ligands to the EV surface by covalent bonds. EV membrane proteins’ amine groups can be converted into alkyne groups and react with azide-tagged ligands via copper-catalyzed azide-alkyne cycloaddition (CuAAC) [87]. For example, the azide-tagged αvβ3 integrin-specific peptide c (RGDyK) [88] and glioma-targeting peptide RGE [87] have been successfully displayed on EV surfaces. However, the critical alkyne modification lacks site specificity control. Thus, click chemistry may jeopardize the structure and function of EV proteins.

Besides, lipids or amphipathic molecules can be inserted into EV lipid bilayer by lipid self-assembly, then tether targeting ligands to EV surface, comprising another chemical strategy [65]. The Polyethylene glycol-grafted 1,2-distearoyl-sn-glycero-3-phosphoethanolamine (DSPE-PEG) has been widely used to anchor targeting ligands on EV membranes for tumor-specific drug delivery since its FDA approval in medical applications [89]. Nevertheless, this method may also elicit higher toxicity of EVs.

#### Hybrid membrane engineering

EVs’ lipid bilayer can spontaneously fuse with other membrane structures, such as synthetic liposomes. For example, Goh et al. have introduced a hybrid system named EXOPLEXs for direct membrane fusion between EVs and liposomes to efficiently deliver large molecules without compromising the EV membrane structure [90]. This hybrid membrane strategy also allowed EV surface modification by fusion with liposomes embedding multiple ligands.

Additionally, the hybridization of EVs with liposomes can be induced by polyethylene glycol (PEG) during freeze and thaw cycles to avoid immune system activation [73, 91]. PEG can hide the hybrid system from immune cells by forming a hydration layer [92]. Therefore, the engineered EVs are endowed with lower immunogenicity, better stability, and prolonged circulation times.

## Advantages of MSC-derived EVs as ideal drug delivery vehicles

### Comparison to EVs derived from body fluids

EVs can be obtained from cell cultures or body fluids, such as blood, saliva, cerebrospinal fluid, urine, semen, and tracheal aspirates [4]. Although biological fluid-derived EVs are promising detection tools for different disease diagnostic biomarkers, cell-derived EVs are nowadays the preferred choice for drug delivery. Upscaling EV production from body fluids is ethnically costly, therefore hard to implement in practice. Besides, body fluid-derived EVs often come from diverse cell types, leading to heterogeneity and interfering with follow-up analysis. For example, serum-derived EVs consist of EVs released by platelets, endothelial cells, and monocytes [27].

### Comparison to EVs derived from other cell sources

All cells can secrete EVs. The most common cell sources include MSCs, immune cells, and cancer cells. Compared with other cell sources, MSCs are the most prolific EV producer and exhibit huge expansion capability for commercially sustainable EV production [6]. Additionally, MSCs can be isolated from different ethically uncontroversial human tissues and have been approved for clinical use by the FDA [93]. Like MSCs, MSC-derived EVs have been demonstrated to exhibit immunosuppressive activity and immunomodulatory properties, which would extend the EV-based drug delivery vehicle's useful live and cargo bioavailability [94]. Increasing clinical evidence has suggested that MSC-derived EVs have good therapeutic effects and are tolerated in different disease animal models without clear adverse effects [95]. Moreover, MSC-derived EVs also display high flexibility for modification and good stability during storage. Regarding other cell sources, current clinical research and applications of immune cell-derived EVs are focusing on their antigen-presenting capacity. They can be used as novel vaccination avenues, carrying intrinsically or extrinsically loaded antigens [96]. Similarly, cancer cell-derived EVs can elicit anti-cancer immune responses by cancer-associated antigen presentation. However, cancer cell-derived EVs can be dangerous because they may carry endogenous oncogenic factors and contribute to cancer [97]. Therefore, MSCs are especially suitable for the mass production of ideal EVs for drug delivery.

### Comparison to other nanocarriers

Unlike traditional nanocarriers (e.g., liposomes), MSC-derived EVs are naturally occurring endogenous vectors with higher biocompatibility and lower immunogenicity [98]. The immunoevasive property of MSC-derived EVs makes it easier for repeated administration because patients would not acquire immunity to the carriers after the first treatment, which currently is a major obstacle to mRNA and gene therapy [99]. Additionally, MSC-derived EVs have better permeability and can freely cross certain biological barriers, such as the blood-retinal and the blood–brain barrier, showing bright prospects for eye and central nervous system diseases treatment [100]. Another significant advantage of MSC-derived EVs is their intrinsic tumor tropism inherited from their parental cells. Their complex surface proteins also provide engineering opportunities to enhance targeting capabilities with exogenous targeting ligands and other surface modification strategies. Instead, liposomes deliver their cargoes mostly through passive accumulation. Also, liposomes’ complex functionalization has failed in clinical trials [101]. Besides, MSC-derived EVs can deliver their cargoes with minimal immune clearance and superior systemic retention in vivo, exhibiting substantial pharmacokinetic benefits [4, 102].

## Applications of bioengineered MSC-derived EVs in cancer therapy

In the previous sections, we reviewed the recent technological progress for drug loading of therapeutic EVs and discussed the advantages of MSC-derived EVs as delivery vehicles. Based on these, in this section, we will detail the current applications of bioengineered MSC-derived EVs in cancer therapy.

### Loading anti-cancer cargoes

As pointed out in this paper introduction, bioengineered MSC-derived EVs possess advantages as delivery vehicles in cancer therapy due to their strong tumor tropism, low immunogenicity, high tolerance, and nanoparticle characteristics [4]. Different anti-cancer cargoes can be packaged into MSC-derived EVs—including miRNAs, anti-miRNAs, siRNAs, mRNAs, drugs, and proteins—through modifications of either parental cells or EVs directly.

#### Nucleic acids

Many studies have shown that transfected MSCs can release EVs encapsulated with specific miRNAs. Once internalized, EVs can deliver miRNAs into cancer cells to regulate tumor development. O'Brien et al. [103] demonstrated that hBMSC-derived EVs loaded with miR-379 suppressed breast cancer via COX-2 regulation. Likewise, miR-146b [104], miR-124a [105], and miR-34a [106] were introduced into glioma cells from transfected hBMSC-derived EVs and abrogated glioma growth by decreasing EGFR and NF-κB protein, silencing FOXA2 and downregulating MYCN, respectively. Other miRNAs have been similarly packed into EVs and worked as anti-cancer agents by post-transcriptional tumor-related gene expression modulation in different cancers [107–117] (summarized in Table4).

**Table 4:** Applications of bioengineered MSC-derived EVs in cancer

EV source	Cancer	Method	Modification	Effect	Proposed mechanism	References
hBMSCs	Breast cancer	In vivo	Loaded miR-379	Tumor growth↓	Regulate COX-2	[103]
rBMSCs	Primary rat astrocytes and glioma	In vivo	Loaded miR-146	Tumor growth↓	EGFR↓ NF-κB↓	[104]
hBMSCs	Glioma	In vitro and in vivo	Loaded miR-124a	Tumor growth↓	FOXA2↓ FOXA2-mediated aberrant intracellular lipid accumulation↑	[105]
hBMSCs	Glioblastoma	In vitro and in vivo	Loaded miR-34a	Proliferation, migration and tumorigenesis↓ Chemosensitivity to TMZ↑	MYCN↓	[106]
hBMSCs	Colorectal cancer	In vitro and in vivo	Loaded miR‐16‐5p	Proliferation, migration, and invasion↓ Apoptosis↑	ITGA2↓	[107]
hBMSCs	Androgen-dependent prostate cancer	In vitro and in vivo	Loaded miR-205	Proliferation, migration, and invasion↓ Apoptosis↑	RHPN2↓	[108]
hUCMSCs	Breast cancer	In vitro and in vivo	Loaded miR-148b-3p	Proliferation, migration, and invasion↓ Apoptosis↑	TRIM59↓ EMT↓	[109]
hUCMSCs	Endometrial cancer	In vitro	Loaded miR-302a	Proliferation↓ Migration↓	Cyclin D1 ↓ AKT signaling pathway↓	[110]
hBMSCs	Pancreatic cancer	In vitro and in vivo	Loaded miR-126-3p	Proliferation, migration, and invasion↓ Apoptosis↑	ADAM9↓	[111]
hBMSCs	Osteosarcoma	In vitro and in vivo	Loaded miR-206	Proliferation, migration, and invasion↓ Apoptosis↑	Target TRA2B	[112]
hBMSCs	Cervical cancer	In vitro and in vivo	Loaded miR-144-3p	Proliferation, migration, and invasion↓ Apoptosis↑	CEP55↓	[113]
hBMSCs	Ovarian cancer	In vitro and in vivo	Loaded miR-424	Proliferation, migration, and invasion↓ Tube formation↓	MYB↓	[114]
hBMSCs	Osteosarcoma	In vitro	Loaded miR-143	Migration↓	N/A	[115]
hBMSCs	Gastric cancer	In vitro	Loaded miR-221	Migration, invasion, and adhesion to the matrix↑	N/A	[116]
hAMSCs	Osteosarcoma	In vitro and in vivo	Loaded miR-101	Migration↓	BCL6↓	[117]
hBMSCs	Breast cancer	In vitro and in vivo	Loaded LNA-anti-miR-142-3p	Apoptosis↑ Tumor growth↓	miR-142-3p↓ miR-150↓ associated tumor suppressor genes including APC and P2X7R↑	[118]
hBMSCs	Pancreatic cancer with Kras^G12D^ mutation	In vitro and in vivo	Loaded KRAS^G12D^ siRNA	Tumor growth↓	CD47-mediated protection; RAS-mediated micropinocytosis	[119]
hBMSCs	Pancreatic cancer	In vitro and in vivo	iEXO-OXA	Tumor growth↓	Tumor-suppressive macrophage polarization, cytotoxic T lymphocytes recruitment and Tregs downregulation	[120]
hAMSCs, hBMSCs, hMenSCs, DPMSCs, hUCMSCs	Prostate tumor; breast adenocarcinoma; Rat glioblastoma	In vitro and in vivo	Loaded mRNA	Apoptosis↑	Intracellular conversion of 5-FC to 5-FU	[121]
mBMSCs	Pancreatic adenocarcinoma	In vitro and in vivo	Loaded Taxol	Tumor growth↓	N/A	[122]
hUCMSCs	Breast cancer; ovarian cancer; lung carcinoma	In vitro and in vivo	Loaded Taxol	Efficient targeting↑ Tumor growth↓ Metastases↓	N/A	[123]
hGinPaMSCs	Pancreatic adenocarcinoma; glioblastoma; mesothelioma; squamous cell carcinoma	In Vitro	Loaded PTX	Tumor growth↓	N/A	[124]
mBMSCs	Mouse colon adenocarcinoma	in Vitro and in vivo	DOX@exosome-apt	Tumor growth↓	N/A	[125]
MSCs	Lung cancer; malignant pleural mesothelioma; renal cancer; breast adenocarcinoma; neuroblastoma	In vitro	TRAIL	Apoptosis↑ Sensitivity to TRAIL↑	N/A	[126]
MSCs	Melanoma; breast adenocarcinoma; lung carcinoma; colon adenocarcinoma	In vitro and in vivo	CTNF-α-exosome-SPIONs	Efficient targeting under an external magnetic field↑ Tumor growth↓ Toxicity↓	Induction of the TNFR I-mediated apoptotic pathway	[127]
hAMSCs	Hepatocellular carcinoma	In vitro and in vivo	Loaded miR-199a	Sensitivity to doxorubicin↑	mTOR pathway↓	[128]
hAMSCs	Hepatocellular carcinoma	In vitro and in vivo	Loaded miR-122	Chemosensitivity↑	Apoptosis and cell cycle arrest↑	[129]
hBMSCs	Glioblastoma multiforme	In vitro	Loaded anti-miR-9	Sensitivity to TMZ↑	Reverse the expression of the multidrug transporter	[130]
hBMSCs	Breast cancer	In vitro and in vivo	Loaded antagomiR222/223	Chemosensitivity↑ Dormancy↓	Regulate cycling quiescence	[131]
hAMSCs	Anaplastic thyroid cancer	In vitro	Loaded TKI	Radioiodine‐sensitivity↑	Thyroid‐specific proteins and transcription factors↑	[132]

*DPMSCs* dental pulp mesenchymal stem cells, *EV* extracellular vesicle, *hAMSCs* human adipose mesenchymal stem cells, *hBMSCs* human bone marrow mesenchymal stem cells, *hGinPaMSCs* human gingival papilla mesenchymal stem cells, *hMenSCs* human menstrual stem cells, *hUCMSCs* human umbilical cord mesenchymal stem cells, *mBMSCs* mouse bone marrow mesenchymal stem cells, *MSC* mesenchymal stem cell, *rBMSCs* rat bone marrow mesenchymal stem cells

Based on the fact that some miRNAs present pro-tumor effects, corresponding inhibitory oligonucleotides can be arranged inside EVs and shuttled into tumor cells to reverse outcomes. For instance, Naseri et al. [118] successfully isolated exosomes from mouse BMSCs and loaded them with locked nucleic acid (LNA)-anti-miR-142-3p by electroporation. The anti-miR-142-3p LNA was delivered to breast cancer cells via exosomes and exhibited anti-tumor effects by miR-142-3p and miR-150 downregulation and subsequently enhancing anti-oncogenes (APC and P2X7R) transcription.

Small interfering RNAs (siRNAs) can also be loaded into exosomes by electroporation. A representative study generated hBMSC-derived exosomes using a bioreactor-based culture system. The exosomes were electroporated with siRNA targeting oncogenic KRAS^G12D^ [119]. The siRNA-exosome-based therapy suppressed Kras^G12D^ mutation pancreatic cancer with enhanced efficacy, both in vitro and in vivo*.* This effect was dependent on CD47-mediated protection and RAS-mediated micropinocytosis [95]. The valuable results have entered the Phase I clinical trial stage. Recently, Zhou et al. reported a significant exosome-based dual delivery biosystem, the iEXO-OXA [120]. In iEXO-OXA, BMSC-derived exosomes were loaded with galectin-9 siRNA by electroporation and with oxaliplatin (OXA) prodrug by surface modification. Once internalized by pancreatic cancer cells, the galectin-9 siRNA blocked the galectin-9/dectin-1 axis to enhance immunotherapy, and the OXA induced immunogenic tumor cell death. Therefore, they collectively suppressed tumor growth in pancreatic cancer.

Besides, mRNA loading of exosomes was investigated by genetic manipulation of parental MSCs. It has been demonstrated that exosomes derived from different MSCs transduced by retrovirus infection with the yCD::UPRT gene could carry the suicide gene mRNA [121]. Induced cell death occurred in the prodrug 5-FC presence by the 5-FC conversion to 5-FU upon suicide gene exosome internalization by tumor cells.

#### Drugs

Similarly, drugs can be incorporated into MSC-derived EVs using pre-loading or post-loading techniques. Regarding pre-loading, Pascucci et al. [122] demonstrated that mouse BMSCs packaged paclitaxel (PTX) after exposure to a very high PTX dosage in vitro for 24 h. They released PTX into tumor cells via their exosomes, leading to tumor growth suppression in pancreatic adenocarcinoma. Melzer et al. [123] also reported a similar approach. They effectively isolated PTX-loaded exosomes from hUCMSC incubated with PTX for 24 h. The PTX-loaded exosomes exhibited tumor growth and metastases inhibitory effects in breast cancer, lung cancer, and ovarian cancer. Similarly, human gingival papilla mesenchymal stem cells (hGinPaMSCs) were primed with a high PTX concentration. Then, the loaded PTX was released and incorporated into cancer cells via EVs to treat human pancreatic carcinoma and squamous carcinoma [124]. In another drug loading strategy, post-loading, the drug is directly packed into EVs after isolating them. For instance, Bagheri et al. [125] used mouse BMSCs-derived exosomes to carry an anti-cancer drug, doxorubicin (DOX), by electroporation as a versatile platform for colorectal cancer treatment.

#### Proteins

Until now, only a few studies have been carried out applying protein-loaded MSC-derived EVs in anti-cancer therapy, among which tumor necrosis factor-related apoptosis-inducing ligand (TRAIL) is representative [126]. TRAIL is a promising anti-cancer protein and possesses the ability to selectively induce cancer cell apoptosis. It has been proved that TRAIL-transduced MSC-derived EVs can express membranal TRAIL. Then, the TRAIL delivery from EVs to cancer cells can induce apoptosis and abolish the TRAIL resistance in lung cancer, malignant pleural mesothelioma, renal cancer, breast adenocarcinoma, and neuroblastoma.

### Improving targeting specificity

Despite native tumor-homing properties, researchers are still exploring new technologies to bring more robust targeting specificity to drug-loaded MSC-derived EVs. Exosome display technology is now widely investigated for exosome targeting by specific ligands attachment to the exosome membrane via surface engineering. Newly introduced membrane ligands endow exosomes with increased tumor tropism and lower systemic toxicity. Exosome membrane ligands’ applications have been reported in many fields. Herein, we describe two applications in MSC-derived exosomes for cancer therapy.

Bagheri’s study [125] (mentioned above) is an example. Before DOX loading, mouse BMSC-derived exosomes were tagged with the 5TR1 aptamer, which has a close affinity with MUC1 protein. MUC1 is an overexpressed transmembrane mucin glycoprotein in some cancer cells. The 5TR1 aptamer was attached to the exosomes' surface by covalent conjugation with surface amine groups via click chemistry. Therefore, the DOX@exosome-apt showed an enhanced tropism and effective inhibition for MUC1-positive cancer cells, providing safe and selective DOX delivery in colon adenocarcinoma.

Additionally, Zhuang et al. reported the CTNF-α-exosome-SPIONs [127] that improved cancer targeting through magnetism and inhibited tumor growth by TNFR I-mediated apoptotic pathway induction. First, exosomes with TNF-α anchored in its membrane (CTNF-α-exosomes) were isolated from MSCs transfected with plasmids encoding CTNF-α. CTNF-α is a fusion peptide consisting of TNF-α and cell-penetrating peptides (CPP). It possesses the lipotropic activity of CPP, enabling TNF-α to anchor in the cell membrane. Next, transferrin-modified superparamagnetic iron oxide nanoparticles (SPIONs) were conjugated to the surface of the CTNF-α-exosomes through transferrin-transferrin receptor interaction. SPIONs were used to deliver drugs to targeted areas by magnetic force. Finally, the CTNF-α-exosome-SPIONs were proved by in vitro and in vivo studies to exert anti-tumor effects under an external magnetic field by efficient TNF-α delivery to cancer cells’ membrane-bound receptors.

### Enhancing chemosensitivity

Besides, modified MSC-derived EVs can be utilized to confer tumor cells chemosensitivity via functional cargo loading and play assistant roles in cancer treatment. For example, miR-199a-transfected hAMSC-derived exosomes elicited enhanced chemosensitivity of hepatocellular carcinoma cells by targeting and subsequently inhibiting the mTOR pathway [128]. A similar effect was reported in miR-122-loaded exosomes from hAMSCs [129]. In another study, results indicated that anti-miR-9 delivery from hBMSC-derived exosomes to glioblastoma multiforme cells sensitized cancer cells to temozolomide [130]. Likewise, Bliss et al. [131] transfected hBMSCs with anti-miR-222/223 and demonstrated the anti-miR-222/223 could be loaded into breast cancer cells via exosomes to increase chemosensitivity. Recently, a new radioiodine-resistant thyroid cancer therapeutic approach via tyrosine kinase inhibitor (TKI)-loaded EVs has been proposed [132]. The TKI was encapsulated into hAMSC-derived EVs by direct incubation or sonication. Packaging efficiency differed, and the sonication was better. The iodine avidity of radioiodine-resistant thyroid cancer cells was abolished after the TKI-loaded EV treatment.

**Fig. 4:**
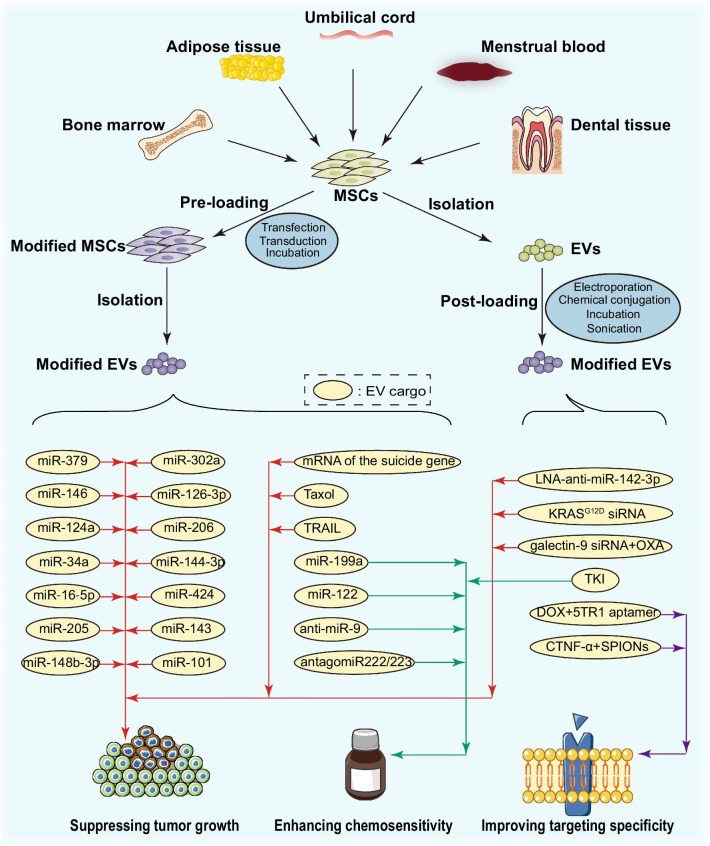
Applications of bioengineered MSC-derived EVs in cancer therapy

Applications of bioengineered MSC-derived EVs in oncology are summarized in Table 4 and Fig. 4. Once internalized by target cells, the modified EVs can release diverse bioactive constituents to regulate target signaling molecules and eventually impair tumor progression. MSC-derived EVs can also be designed to enhance targeting specificity, safety, and efficiency, being a promising therapeutic delivery vehicle. However, each modification method has its pros and cons. Indeed, EV bioengineering strategies are rapidly developing, and new technologies are emerging for different disease treatments with various parental cells, not limited to MSCs [133, 134]. In the future, these advanced technologies should also be used in MSC-derived EV-based anti-cancer agent delivery system.

## Future challenges and directions

Clinical-grade MSC-derived exosomes encapsulated with Kras^G12D^siRNA have been used to treat pancreatic cancer in multiple animal models, increasing mice’s overall survival without any clear toxicity and improving targeting specificity [95, 119]. Further investigation of the KRAS^G12D^ siRNA-loaded exosome-based therapy has entered Phase I clinical trial for Kras^G12D^ mutation pancreatic cancer treatment. So far, 17 clinical trials using MSC-derived EVs as therapeutic avenues have been registered (listed in www.clinicaltrials.gov) [135] (Table 5). However, only one of them focuses on cancer treatment and few results are available. Many hurdles slow down the fledgling clinical utilization of MSC-derived EVs.

**Table 5:** Registered clinical trials involving MSC-derived EVs

Identifier	Disease	EV source	Key cargo	Status	Year of registration
NCT03384433	Acute ischemic stroke	MSCs	miR-124	Recruiting	2017
NCT03437759	Macular holes	UCMSCs	N/A	Active, not recruiting	2018
NCT03608631	Metastatic pancreas cancer with Kras^G12D^ mutation	BMSCs	KRAS^G12D^ siRNA	Recruiting	2018
NCT03857841	Bronchopulmonary Dysplasia	BMSCs	N/A	Active, not recruiting	2019
NCT04134676	Chronic Ulcer	WJ-MSCs	N/A	Completed	2019
NCT04173650	Dystrophic Epidermolysis Bullosa	BMSCs	N/A	Not yet recruiting	2019
NCT04213248	Dry eye related to cGVHD	UCMSCs	N/A	Recruiting	2019
NCT04223622	Osteoarthritis	AMSCs	N/A	Not yet recruiting	2020
NCT04276987	Severe novel coronavirus pneumonia	AMSCs	N/A	Completed	2020
NCT04356300	MODS after surgical repaire of ATAAD	UCMSCs	N/A	Not yet recruiting	2020
NCT04388982	Alzheimer's Disease	AMSCs	N/A	Recruiting	2020
NCT04491240	SARS-CoV-2 Associated Pneumonia	MSCs	N/A	Completed	2020
NCT04544215	Carbapenem-resistant gram-negative bacilli-induced pulmonary infection	AMSCs	N/A	Recruiting	2020
NCT04602104	ARDS	MSCs	N/A	Not yet recruiting	2020
NCT04602442	COVID-19 Associated Pneumonia	MSCs	N/A	Enrolling by invitation	2020
NCT04657458	COVID-19 associated ARDS	BMSCs	N/A	Available	2020
NCT04798716	ARDS or NCP caused by COVID-19	MSCs	N/A	Not yet recruiting	2021

*AMSCs* adipose mesenchymal stem cells, *ARDS* acute respiratory distress syndrome, *ATAAD* acute type A aortic dissection, *BMSCs* bone marrow mesenchymal stem cells, *cGVHD* chronic graft versus host diseases, *EV* extracellular vesicle, *MODS* multiple organ dysfunction syndrome, *MSC* mesenchymal stem cell, *NCP* novel coronavirus pneumonia, *UCMSCs* umbilical cord mesenchymal stem cells, *WJ-MSCs* Wharton's Jelly mesenchymal stem cells

### Safety

The dual roles of MSC-derived EVs in oncogenesis, tumor progression, and chemoresistance are highly variable, depending on MSC origins and tumor types. Therefore, the native MSC-derived EVs’ safety controversy has long prevailed and is regarded as their “Achilles’ heel” for clinical applications. The investigation into the impact of one EV type on one specific cancer and their mechanisms are necessary. Thus, the most appropriate MSC source might be screened for bioengineered EV production to treat the specific cancer type with fewer adverse effects. On the other hand, it is also needed to apply the same type of EVs to different cancers to determine the potential therapy scope. The development of methods to deactivate or remove unwanted and harmful EV contents may be a significant and novel engineering strategy. Moreover, MSC-derived EVs’ long-term safety and therapeutic effects should be verified by future follow-ups. A monitoring platform in vivo is also required to obverse drug distribution, optimize dosage regimens, and guarantee therapeutic safety [3].

### Efficiency

A second limitation of MSC-derived EV-based treatments is the heavy workload but low yield during production. Additionally, unsatisfactory drug loading and delivery efficiency seems to be common problems in all EV-related clinical applications. Their clinical breakthrough highly hinges on nanotechnology and genetic engineering advances.

During the past ten years, exosome-mimetics (EMs) have become prominent new drug delivery systems. They are bioinspired and synthetically personalized nanovesicles with similar characteristics and therapeutic effects of EVs [136, 137]. Unlike EVs, EMs can be produced on a much larger scale by membrane filter extrusion, pressurization or slicing over microfluidic devices, and hybrid biomimicry strategies [138]. Interested readers might refer to Antimisiaris [139] and Lu and Huang [138] for more detailed reviews on EM technologies and applications. Implementing MSCs-derived EMs in cancer treatment can be a future direction. For example, Kalimuthu et al. [140] have isolated EMs from hBMSCs mixed with PTX by extrusion and demonstrated their significant therapeutical effects against breast cancer. Similarly, EMs isolated from human induced pluripotent stem cells (iPSCs)-derived MSCs provided efficient DOX and docetaxel delivery to triple-negative breast cancer [141] and metastatic prostate cancer [142].

Besides, the therapeutical efficiency also depends on MSCs’ availability and expandability. Currently, bone marrow MSCs are the most frequently used, followed by the umbilical cord MSCs and adipose MSCs. In further research, menstrual blood MSCs and dental tissue MSCs deserve more attention due to their convenient and noninvasive accessibility. Concerning expandability, human induced pluripotent stem cells (iPSCs) have been used to produce MSCs with limitless expandability, in theory [143–145]. Increasing efforts are still required to ensure the efficacy of EVs derived from these MSCs.

### Standardization

A recently published paper presented isolation and characterization protocols for six different EV subpopulations from tissues [146]. However, EV classification has not yet been unified. Definitions such as extracellular vesicles, microvesicles, and exosomes are obscure and inconsistent among past studies. Additionally, some findings may be derived from several heterogeneous subpopulations [4]. Further research should distinguish different MSC-derived EV subpopulations and elucidate their respective roles in cancer development. A more comprehensive understanding of intercellular communications between cancer cells and MSC-derived EVs may also provide novel insights into cancer biology and pave the way for MSC-derived EV-based drug delivery systems. Considering EVs’ functional complexity and heterogeneity, there is an urgent need to establish refined systematic standards for the culture conditions, modification, production, purification, characterization, and storage of bioengineered MSC-derived EVs before clinical applications.

## Conclusions

Overall, MSC-derived EVs can present multiple effects on tumor development and serve as promising anti-tumor drug delivery platforms due to their strong tumor tropism. However, the utilization of MSC-derived EVs in cancer treatment is still at the beginning. Further studies are required to accelerate their therapeutic clinic application.

## Data Availability

Not applicable.

## Abbreviations

5-FC: 5-Fluorocytosine
5-FU: 5-Fluorouracil
AFM: Atomic force microscopy
AMSCs: Adipose mesenchymal stem cells
ARDS: Acute respiratory distress syndrome
ATAAD: Acute type A aortic dissection
BMSCs: Bone marrow mesenchymal stem cells
cGVHD: Chronic graft versus host diseases
CIB1: Basic-helix-loop helix 1
CPP: Cell-penetrating peptides
CRY2: Photoreceptor cryptochrome 2
CuAAC: Copper-catalyzed azide-alkyne cycloaddition
DLS: Dynamic light scattering
DOX: Doxorubicin
DPMSCs: Dental pulp mesenchymal stem cells
DSPE: 1,2-Distearoyl-sn-glycero-3-phosphoethanolamine
EM: Electron microscopy
EMs: Exosome-mimetics
EMT: Epithelial-mesenchymal transition
ER: Endoplasmic reticulum
ESE: Early-sorting endosome
EV: Extracellular vesicle
FC: Flow cytometry
GNSTM: Glycosylation motif
GPI: Glycosylphosphatidylinositol
hAMSCs: Human adipose mesenchymal stem cells
hBMSCs: Human bone marrow mesenchymal stem cells
HGF: Hepatocyte growth factor
hGinPaMSCs: Human gingival papilla mesenchymal stem cells
hMenSCs: Human menstrual stem cells
hUCMSCs: Human umbilical cord mesenchymal stem cells
ILV: Intraluminal vesicle
iPSCs: Induced pluripotent stem cells
ISEV: International Society for Extracellular Vesicles
Lamp2b: Lysosomal-associated membrane protein 2
LNA: Locked nucleic acid
LSE: Late-sorting endosome
mBMSCs: Mouse bone marrow mesenchymal stem cells
MET: Mesenchymal–epithelial transition
MODS: Multiple organ dysfunction syndrome
MSC: Mesenchymal stem cell
MVB: Multivesicular body
NCP: Novel coronavirus pneumonia
NTA: Nanoparticle tracking analysis
OXA: Oxaliplatin
PDGFR: Platelet-derived growth factor receptor
PEG: Polyethylene glycol
PTX: Paclitaxel
rBMSCs: Rat bone marrow mesenchymal stem cells
ROS: Reactive oxygen species
RVG: Rabies viral glycoprotein
SEM: Scanning electron microscopy
siRNAs: Small interfering RNAs
SPIONs: Superparamagnetic iron oxide nanoparticles
TEM: Transmission electron microscopy
TKI: Tyrosine kinase inhibitor
TRAIL: Tumor necrosis factor-related apoptosis-inducing ligand
TRPS: Tunable resistance pulse sensing
UCMSCs: Umbilical cord mesenchymal stem cells
WJ-MSCs: Wharton's Jelly mesenchymal stem cells
